# Sensory-thresholded switch of neural firing states in a computational model of the ventromedial hypothalamus

**DOI:** 10.3389/fncom.2022.964634

**Published:** 2022-09-07

**Authors:** Ryan Rahy, Hiroki Asari, Cornelius T. Gross

**Affiliations:** Epigenetics and Neurobiology Unit, European Molecular Biology Laboratory (EMBL), Monterotondo, Italy

**Keywords:** ventromedial hypothalamus (VMH), fear, avoidance (withdrawal), feedback, inhibition, neuromodulation, modeling, computational

## Abstract

The mouse ventromedial hypothalamus (VMH) is both necessary and sufficient for defensive responses to predator and social threats. Defensive behaviors typically involve cautious approach toward potentially threatening stimuli aimed at obtaining information about the risk involved, followed by sudden avoidance and flight behavior to escape harm. *In vivo* neural recording studies in mice have identified two major populations of VMH neurons that either increase their firing activity as the animal approaches the threat (called Assessment+ cells) or increase their activity as the animal flees the threat (called Flight+ cells). Interestingly, Assessment+ and Flight+ cells abruptly decrease and increase their firing activity, respectively, at the decision point for flight, creating an escape-related “switch” in functional state. This suggests that the activity of the two cell types in VMH is coordinated and could result from local circuit interactions. Here, we used computational modeling to test if a local inhibitory feedback circuit could give rise to key features of the neural activity seen in VMH during the approach-to-flight transition. Starting from a simple dual-population inhibitory feedback circuit receiving repeated trains of monotonically increasing sensory input to mimic approach to threat, we tested the requirement for balanced sensory input, balanced feedback, short-term synaptic plasticity, rebound excitation, and inhibitory feedback exclusivity to reproduce an abrupt, sensory-thresholded reciprocal firing change that resembles Assessment+ and Flight+ cell activity seen *in vivo*. Our work demonstrates that a relatively simple local circuit architecture is sufficient for the emergence of firing patterns similar to those seen *in vivo* and suggests that a reiterative process of experimental and computational work may be a fruitful avenue for better understanding the functional organization of mammalian instinctive behaviors at the circuit level.

## 1. Introduction

A major goal of current neuroscience research is to arrive at a circuit-based understanding of behavior so as to be able to better describe and categorize the origins of animal behavior and rationally design behavior-modifying treatments. The last decade has seen an enormous advance in the tools available to behavioral circuit neuroscientists who can now record hundreds of neurons simultaneously in freely behaving laboratory animals and activate or inhibit the firing of defined classes of neurons at the millisecond timescale (Yizhar et al., 2011). These tools have ushered in an era in which new approaches are needed to understand the link between neural firing activity and behavior, and there has been a renewed call to engage with theoretical neuroscientists to build predictive models and derive testable hypotheses. Ideally, such collaborations between experimentalists and theoreticians would fuel a virtuous experiment-theory cycle that could help arrive at an understanding of the circuit basis of behavior (Marr and Poggio, 1976; Yuste, 2008).

The medial hypothalamic defensive system offers a fertile subject for circuit behavior neuroscience because it consists of a series of interconnected nuclei that are anatomically and functionally conserved across mammals, including primates, Canteras (2002) and Montardy et al. (2021) and it forms a relatively simple subcortical pathway linking sensory input to motor output (Canteras, 2002; Gross and Canteras, 2012). Extensive work has been carried out on a central node in this system, the ventromedial hypothalamus (VMH), and inhibition of this nucleus blocks defensive responses to predator and social threats (Silva et al., 2013; Kunwar et al., 2015), while activation elicits flight behavior (Lipp and Hunsperger, 1978; Hess and Brügger, 1981; Lin et al., 2011; Kunwar et al., 2015; Sakurai et al., 2016; Krzywkowski et al., 2020). Consistent with its anatomical conservation between rodents and primates (Lipp and Hunsperger, 1978; Montardy et al., 2021) electrical stimulation of VMH in humans elicits intense arousal, negative emotion, and panic, Wilent et al. (2010) supporting the hypothesis that VMH encodes an internal defensive state that is relevant to understanding instinctive fear in humans (Anderson, 2016).

*In vivo* single unit electrophysiology and calcium endoscopy recordings in freely behaving mice exposed to predator or social threat have revealed that VMH encodes both sensory and motor features of defensive behavior (Krzywkowski et al., 2020; Masferrer et al., 2020). Single unit electrophysiology recordings were carried out in VMH as mice approached and then fled a live rat, a natural predator. Twenty-eight percent of units showed significant changes in firing during the approach-to-flight behavior. Of these, 39% showed either increased or decreased firing during approach—called Assessment+ and Assessment- cells, respectively—while 61% showed either increased or decreased firing during flight - called Flight+ and Flight- cells, respectively (Masferrer et al., 2020). Notably, Assessment+ cells abruptly decreased firing at the moment in which the animal initiated escape, while Flight+ cells abruptly increased firing at this point. These data demonstrate that separate neuron populations in VMH encode sensory information about threat and motor information about the defensive behavioral outcome and suggest that a transition from sensory to motor encoding occurs immediately prior to flight. Moreover, because the firing rate of Assessment+ cells increased linearly as the animal approached the threat (Masferrer et al., 2020) the abrupt change in firing of Assessment+ and Flight+ cells appeared to occur in a manner whose probability was increased by or thresholded to the intensity of threat exposure (Supplementary Figure 1). While these recordings were carried out in the dorsomedial VMH (VMHdm) during exposure of mice to a rat, similar Assessment+ and Flight+ cells were seen in the neighboring ventrolateral VMH (VMHvl) during exposure of mice to a social aggressor, Krzywkowski et al. (2020) consistent with the anatomical segregation of predator and social threat processing (Motta et al., 2009; Gross and Canteras, 2012; Silva et al., 2013; Wang et al., 2019) and suggesting a generalized sensory-motor encoding of innate defensive responses across threats in the medial hypothalamus.

Two previous studies have reported computational models of VMH (MacGregor and Leng, 2019; Kennedy et al., 2020). The former was based on a classification of neurons with putative differences in intrinsic excitability based on hazard plots of firing propensity extracted from *in vivo* single unit recordings of VMHdm neurons in anesthetized rats (Sabatier and Leng, 2008). These excitability properties were modeled in a network of interconnected excitatory neurons to derive network firing activities. The study concluded that slow oscillatory rhythms and network bistability similar to those experimentally observed could emerge from a model of a heterogeneous population of VMH neurons. Interestingly, the model revealed subclasses of cells that showed spontaneous ON/OFF firing states, pointing to an intrinsic propensity for sudden state transitions among VMH neurons (MacGregor and Leng, 2019). The second study constructed a circuit model of the mouse VMHdm composed of interconnected excitatory core neurons receiving sensory input and inhibitory feedback (Kennedy et al., 2020). The study aimed to identify conditions under which persistent neural firing activity emerged in response to transient sensory input. Such persistent activity was proposed as a substrate for an internal defensive state in VMH deriving from experimental (Kunwar et al., 2015) and theoretical (Anderson and Adolphs, 2014) considerations. The model showed that the addition of slow-acting neuromodulatory transmission between VMH core neurons led to sustained bulk activity of core neurons. Interestingly, in addition to sensory-ON neurons whose activity matched sensory input, neurons with sensory-OFF properties were observed that showed peak activity at stimulus offset, demonstrating that the combination of excitatory interconnectivity and feedback inhibition could give rise to neuron classes with reciprocal sensory-ON/OFF stimulus responses.

We explored whether a computational model of local VMHdm circuitry could give rise to the transition in firing state observed during approach-to-flight behavior (Masferrer et al., 2020) and be used to constrain future experimental circuit manipulation studies. We aimed to produce a parsimonious model in which three firing features would emerge: (1) distinct Assessment+ and Flight+-like cell populations, (2) a sensory-input thresholded abrupt change in global firing state, and (3) a simultaneous and opposite change in firing activity of Assessment+ and Flight+-like cells at threshold. In other words, a reciprocal “switch” in activity of Assessment and Flight cell populations should occur when sensory input passes a specific level. Our findings show that thresholded switching between Assessment and Flight-like populations can occur under conditions of excitatory neuron interconnectivity coupled to feedback inhibition with neuromodulatory rebound excitation, a feature observed in *in vivo* neural recordings (Wong et al., 2016). These conditions set out several non-obvious hypotheses for experimental testing using circuit recording and manipulation tools in behaving animals.

## 2. Materials and methods

### 2.1. Circuit model

To identify key network features underlying a sensory-thresholded switch between Assessment+ and Flight+ cells we built circuit models in the NEST Simulator (Fardet et al., 2020, RRID:SCR_002963) consisting of three major cell types (Amygdala, VMH core, VMH shell) and introduced different network geometries and properties among them. All models used leaky integrate-and-fire neurons with exponentially-shaped postsynaptic currents (Tsodyks et al., 2000) and Bernoulli synapses (static synapses with release probability = *p*_*release*_), with a timestep of 0.1 ms. All connections within and between populations followed a Bernoulli distribution (connection probability = *p*_*connection*_). VMH core included two populations of excitatory neurons (*N* = 100 each, Assessment and Flight neurons). Parameters were chosen based on previously reported *in vitro* slice electrophysiological properties of VMH core and shell neurons (core: resting membrane potential = −58.6 mV, action potential threshold = −43.1 mV, membrane time constant = 40.3 ms; shell: resting membrane potential = −56.5 mV, action potential threshold = −42 mV, membrane time constant = 41.6 ms) (Yamamoto et al., 2018). VMH shell included either two populations (Models 1–3: *N* = 20 each, exclusive Assessment-to-Flight/Flight-to-Assessment connectivity) or a single population (Model 4: *N* = 40 neurons, random Assessment-to-Flight/Flight-to-Assessment connectivity). Shell neurons are predisposed to having higher firing rates (Yamamoto et al., 2018) and were given higher noise frequencies than excitatory core neurons (core: 5 Hz; shell: 10 Hz). Where relevant, imbalanced weights of shell-to-Assessment and shell-to-Flight connections were set by multiplying the inhibitory weight of selected connections by a ratio 1 ≤ *r*_*feedback*_ ≤ 20 (Model 1, Hybrid Model). The Amygdala consisted of a uniform population of non-interconnected excitatory neurons (*N* = 100) receiving sensory input and asymmetrically projecting onto both VMH core populations with varying connection density to Flight neurons obtained by multiplying the Amygdala-to-Flight *p*_*connection*_ by a ratio 0 ≤ *r*_*input*_ ≤ 1 (Models 1–4). Weights of Amygdala connections to VMH core were sampled randomly from a normal distribution, while delay values were sampled from a uniform distribution to introduce variability in the input received by VMH core. All parameter values used are listed in Supplementary Table 1. We aimed to make these values as consistent as possible across models, but due to differing model features and synaptic properties some variation was necessary.

### 2.2. Sensory input

To simulate the sensory input that a mouse experiences during approach to and flight away from a predator we employed an inhomogeneous Poisson process that generated spiking signals to the Amygdala population based on a time-varying firing rate function λ(*t*). In each trial (20 s) we used λ(*t*) = *max*(*f*_*baseline*_, *a*_*model*_*t*) for the first half of the trial (0 < *t* <10 *s*) to approximate the increasing sensory signal during approach behavior (assessment phase), and λ(*t*) = *max*[*f*_*baseline*_, *f*_*max, model*_−4*a*_*model*_(*t*−10)] for the remaining 10 s (10 < *t* <20 *s*) to simulate the decreasing sensory signal during avoidance behavior (flight phase), where *f*_*baseline*_ = 40 *Hz* sets the baseline level of input to the Amygdalar cells, *f*_*max, model*_ represents the maximum sensory input firing rate for each model, and $a_{model}=\frac{f_{max,\;model}}{10}$ is the slope for each model. The values for each *f*_*max, model*_ can be found in Supplementary Table 1. Note that a mouse typically flees faster than it approaches a threat, hence the rapid drop in sensory input (Supplementary Figure 1; Masferrer et al., 2020). We included multiple trials in a single simulation run to assess consistency in the network response without resetting parameters. Each run of our simulation included 10 consecutive trials with inter-trial intervals of 6 s and buffer periods of 2 s at the beginning and end. During the inter-trial intervals and buffer periods we used λ(*t*) = *f*_*baseline*_ to represent a baseline level of sensory input to the Amygdala.

### 2.3. Synaptic plasticity and rebound

Short-term plasticity was implemented in core-efferent synapses in Model 2 and the Hybrid Model as described in the Tsodyks-Markram synaptic model (Tsodyks et al., 2000). Briefly, this model reproduces the effect of plasticity by increasing the release probability of a given synapse by a certain increment with each presynaptic spike (facilitation time constant, 0 ≤ τ_*facilitation*_ ≤ 10, 000 *ms*, reflects how fast the synapse returns to the initial release probability). It also decreases the synaptic resources after each spike (depression time constant, 0 ≤ τ_*depression*_ ≤ 10, 000 *ms*, reflects the rate of synaptic vesicle replenishment). Because NEST does not allow for having both fast and slow transmission in the same synapse, excitatory rebound was introduced into Models 3 and 4 by adding an intermediate population of neurons between shell and core with non-probabilistic release synapses and a slow excitatory effect (delay time, 0 ≤ *t*_*delay*_ ≤ 50 *ms*; rebound time constant, 0 ≤ τ_*rebound*_ ≤ 50 *ms*; estimated from experimental data: *t*_*delay*_ = 14 *ms*, τ_*rebound*_ = 10 *ms*; Wong et al., 2016).

### 2.4. Model feature analysis

Parameter exploration of model-specific features was performed by running each model over single-trial-per-run simulations with different values for the key parameters of each model within specified ranges. Each simulation was then classified qualitatively; a simulation was classified as a “Switch” if Assessment and Flight firing patterns satisfied the following criteria: (1) Assessment onset was gradual, decrease was sudden, (2) Flight onset was sudden, decrease was gradual, (3) Firing was reciprocal—Flight onset reduced Assessment firing to at least 40% of maximum, (4) Signals were sustained, but Flight firing returned to baseline within trial time, and (5) Assessment firing did not go up beyond baseline levels after Flight firing stopped. Simulations that did not satisfy all five criteria were classified as an “Intermittent reciprocal firing simulation” where Assessment and Flight neurons showed unstable, repeated reciprocal changes in firing activity, or “None” when the simulation could not be placed in any other category. “None” simulations were not represented in the scatterplots of the parameter space. For parameter spaces with more than three dimensions, principal component analysis (PCA) was used to represent the results in three dimensions. To test for thresholding of sensory input, we ran each model over simulations with 10 trials per run and varying slopes of sensory input. For each trial, the latency was calculated as the time from the start of the increase in sensory input to the onset of the switch. An inverse correlation between latency and sensory input at the onset of the switch was interpreted as a sign of switch thresholding to a certain level of sensory input.

### 2.5. Analysis of firing activity

All analyses of neuron firing were carried out in Python using the PySpike package (Mulansky and Kreuz, 2016). For population analysis we first computed the peri-stimulus time histogram (PSTH; bin size = 20 ms) of all cells combined for each population (e.g., all Assessment or all Flight neurons). For each of the 10 trials we determined the onset time of the switch by identifying the moment at which the firing of the Flight population surpassed that of the Assessment population by more than 25% of the overall PSTH maximum of both populations. Finally, we averaged maximum-normalized 20-s windows of each population PSTH centered around each putative switch. For single neuron analysis we repeated the same procedure using the firing activity of individual cells.

## 3. Results

### 3.1. Circuit model architecture

We argued that a successful computational model of VMHdm should lead to the emergence of two distinct neuron populations showing an abrupt reciprocal change in activity at a sensory input threshold—corresponding to Assessment+ and Flight+ neuron activity reported during approach-to-flight behavior (Supplementary Figure 1; Masferrer et al., 2020). To explore the connectivity, intrinsic excitability, and synaptic plasticity conditions under which such a sensory-thresholded, reciprocal neuronal response might emerge, we implemented a circuit model of leaky integrate-and-fire neurons in the NEST computing environment (Fardet et al., 2020). The VMHdm consists of a mainly excitatory core with a predominantly inhibitory shell, with synaptic connections between and within core and shell neurons (Yamamoto et al., 2018; Kim et al., 2019; Kennedy et al., 2020). Our model therefore consisted of a group of interconnected excitatory core VMH neurons (*N* = 200) segregated into two populations, a priori called Assessment and Flight cells (*N* = 100 each), so as to be able to vary their physiological characteristics separately, if needed. These neurons received reciprocal inhibitory feedback *via* shell VMH inhibitory neurons (*N* = 40) and sensory input from excitatory Amygdala neurons (*N* = 100). Previous work showed that amygdala inputs to VMH convey predator and social threat information (Swanson and Petrovich, 1998; Petrovich et al., 2001), including pheromone/kairomone-based information about threat identity (Canteras et al., 1995; Lin et al., 2011; Li et al., 2017; Miller et al., 2019) and polymodal information about threat intensity (Canteras et al., 1992; Petrovich et al., 1996; Stagkourakis et al., 2020; Yamaguchi et al., 2020; Zha et al., 2020). Because Assessment+, but not Flight+ neuron firing *in vivo* correlated linearly with inverse distance to threat—and thus to the intensity of threat-related sensory input (Masferrer et al., 2020)—we explored both the simple condition under which Assessment, but not Flight cells received sensory input from Amygdala, and also conditions under which Assessment and Flight cells received varying relative Amygdala inputs (Flight = 0 to 100% Assessment input; see Section 2). We note that due to a lack of precise information about the nature of the threat information carried by anatomical inputs to VMH, amygdala inputs to VMH were approximated by a single excitatory afferent population. Moreover, throughout our study sensory input aimed at representing approach-to-flight behavior was modeled by repeated trains of brief, linearly increasing, and noisy excitatory input to Amygdala cells (Supplementary Figure 2).

### 3.2. Model 1: Asymmetric feedback inhibition

**Figure 1:**
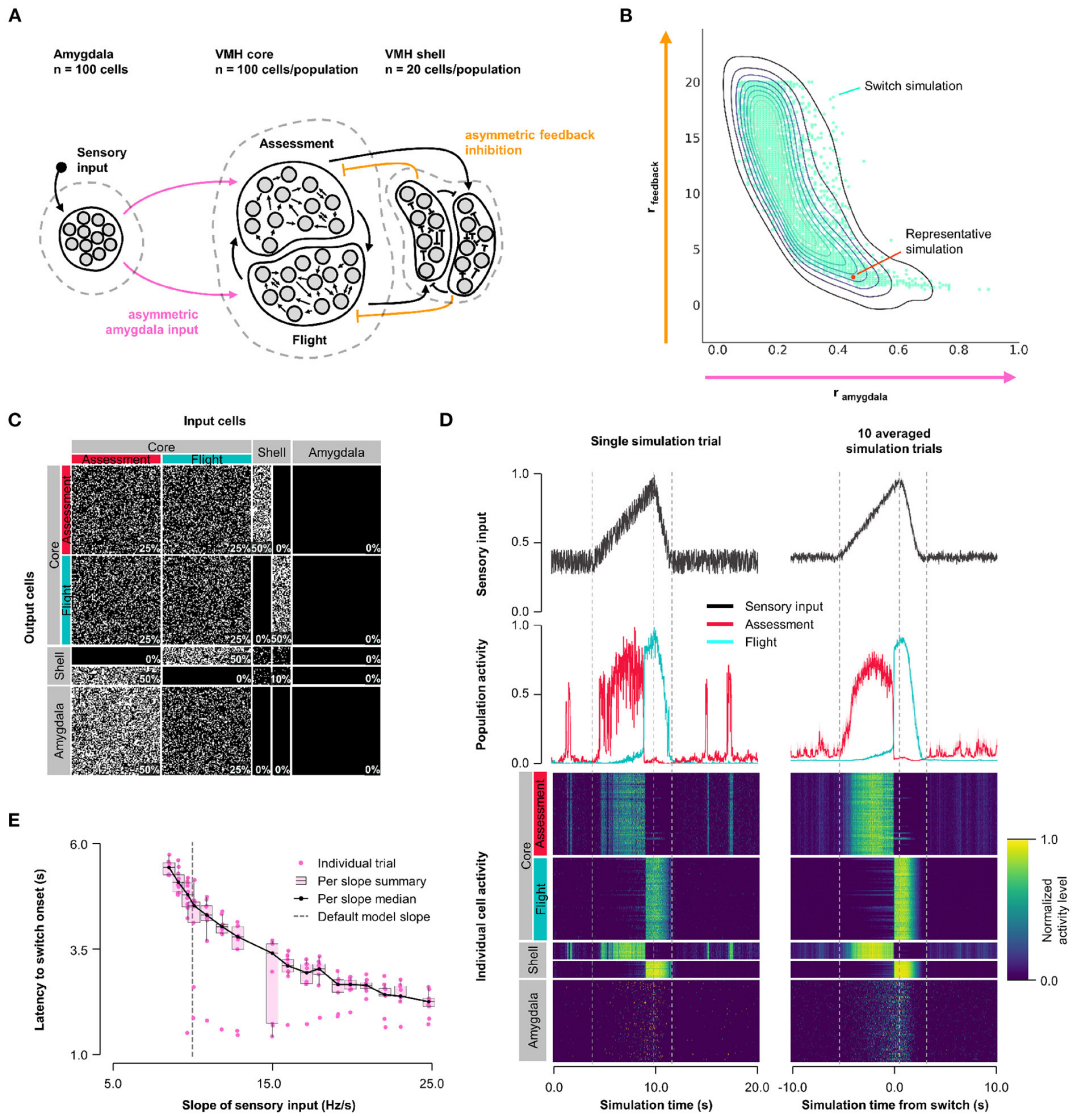
Model 1. Asymmetric feedback inhibition. **(A)** Circuit structure in which both the ratio (*r*_*input*_) of the probability of Amygdala-Flight/Amygdala-Assessment connectivity (pink) as well as the ratio (*r*_*feedback*_) of the weights of shell-Assessment/shell-Flight connectivity (orange) were systematically varied. **(B)** Systematic model exploration identified parameters that satisfy criteria for Assessment/Flight switching (turquoise, see Section 2), where only simulation parameters that satisfy these criteria are shown. A representative switch simulation is highlighted in orange. **(C)** Network connectivity matrix of the representative simulation showing connectivity probabilities between all neuron populations. **(D)** Network firing activity from the representative simulation for a single-trial simulation run (left) or a 10-trial averaged simulation run (right). Top row shows sensory input, middle row shows firing patterns of Assessment (red) and Flight (blue) populations, and bottom row shows firing activity of all individual cells, separated by population. All trials were centered around the time the switch occurs before averaging. The vertical dashed lines mark the beginning and end of the assessment and flight phases of the sensory input (see Section 2). **(E)** Plot of sensory input slope vs. latency of switching showing an inverse relationship between latency and sensory threshold. Only slope values for which a switch was observed are indicated.

First, we explored how the balance of feedback inhibition onto Assessment and Flight cells affected VMH core neuron responses to sensory input (Model 1, Figure 1A). A systematic assessment of core neuron sensory responses under conditions of varying Amygdala input density (0 ≤ *r*_*input*_ ≤ 1) and feedback inhibition strength (1 ≤ *r*_*feedback*_ ≤ 20) revealed parameters under which a simultaneous and opposite change in firing activity of Assessment and Flight neuron sensory responses emerged—a condition we refer to as a “switch” in activity—when Assessment cells received denser sensory input (*r*_*input*_∈[0.2, 0.8]) and stronger feedback inhibition (*r*_*feedback*_∈[2, 20]) than Flight cells. This finding argues that intrinsic differences in the connectivity of VMH cell populations may be necessary for differential Flight and Assessment responses to sensory input (Figure 1B, Supplementary Figure 3A; representative simulation: *r*_*input*_ = 0.5, *r*_*feedback*_ = 2.5, Figures 1C,D). Importantly, the switch in Assessment and Flight neuron firing occurred during the increasing phase of sensory input suggesting that the switch may be thresholded to a specific level of sensory input. Consistent with such sensory input thresholding, the latency to switch onset varied inversely with sensory input slope (Figure 1E).

### 3.3. Model 2: Short-term plasticity

**Figure 2:**
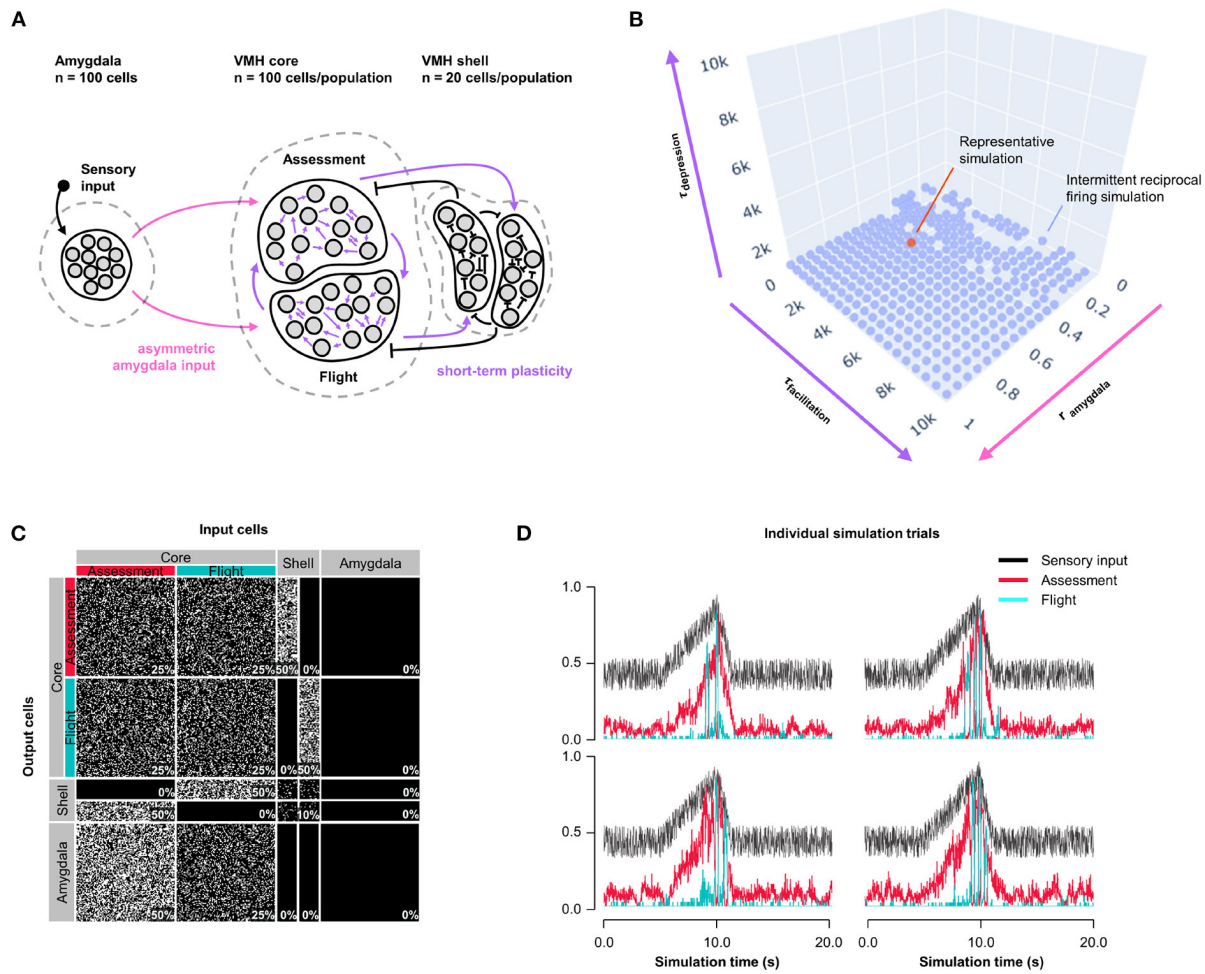
Model 2: Short-term plasticity. **(A)** Circuit structure in which both depression and facilitation time constants for short-term plasticity introduced at all excitatory core output connections (purple) as well as the ratio (*r*_*input*_) of the probability of Amygdala-Flight/Amygdala-Assessment connectivity (pink) were systematically varied. **(B)** Systematic model exploration failed to identify parameters that consistently produced a stable switch. Instead, model parameters that showed intermittent reciprocal Assessment/Flight activity are indicated (blue), as is a representative reciprocal simulation (orange). **(C)** Network connectivity matrix of the representative simulation showing connectivity probabilities between all neuron populations. **(D)** Network firing activity from the representative simulation of a single 10-trial simulation run showing six individual trials with unstable and inconsistent firing patterns.

Because Assessment/Flight responses in Model 1 remained relatively gradual compared to the abrupt reciprocal change in firing rates seen *in vivo* (Supplementary Figure 1) we explored whether the inclusion of synaptic plasticity might convey a sharpening of the switch. Short-term synaptic plasticity can have a presynaptic or postsynaptic origin and is a short-lasting non-linear neurotransmission mechanism described across many neuronal connections (Fioravante and Regehr, 2011) including amygdala inputs to VMH (Stagkourakis et al., 2020). We added short-term, pre-synaptic, facilitating plasticity to all core neuron outputs, including both core-core and core-shell synapses, in a model with varying Amygdala input density and symmetric feedback inhibition strength and examined Assessment and Flight neuron responses (Model 2, Figure 2A). A systematic exploration of synaptic plasticity parameters (0 ≤ τ_*facilitation*_ ≤ 10, 000 *ms*; 0 ≤ τ_*depression*_ ≤ 10, 000 *ms*) and Amygdala input asymmetry (0 ≤ *r*_*input*_ ≤ 1) failed to identify parameters that gave rise to a stable switch in Assessment and Flight neuron responses (Figure 2B; representative simulation: τ_*facilitation*_ = 3, 158 *ms*, τ_*depression*_ = 527 *ms*, *r*_*input*_ = 0.5, Figures 2C,D). Under these conditions Assessment neurons showed sensory-ON responses but Flight neurons exhibited intermittent firing responses that failed to show a consistent pattern across trials. Nevertheless, the introduction of short-term synaptic plasticity was able to sharpen reciprocal Assessment/Flight neuron activity changes as Flight neuron firing was associated with rapid suppression of Assessment neurons. Interestingly, unlike in Model 1, this behavior was independent of Amygdala input asymmetry (Supplementary Figure 3B) showing that abrupt reciprocal Assessment/Flight firing changes can occur even when they have indistinguishable sensory inputs.

### 3.4. Hybrid model: Asymmetric feedback inhibition and short-term plasticity

**Figure 3:**
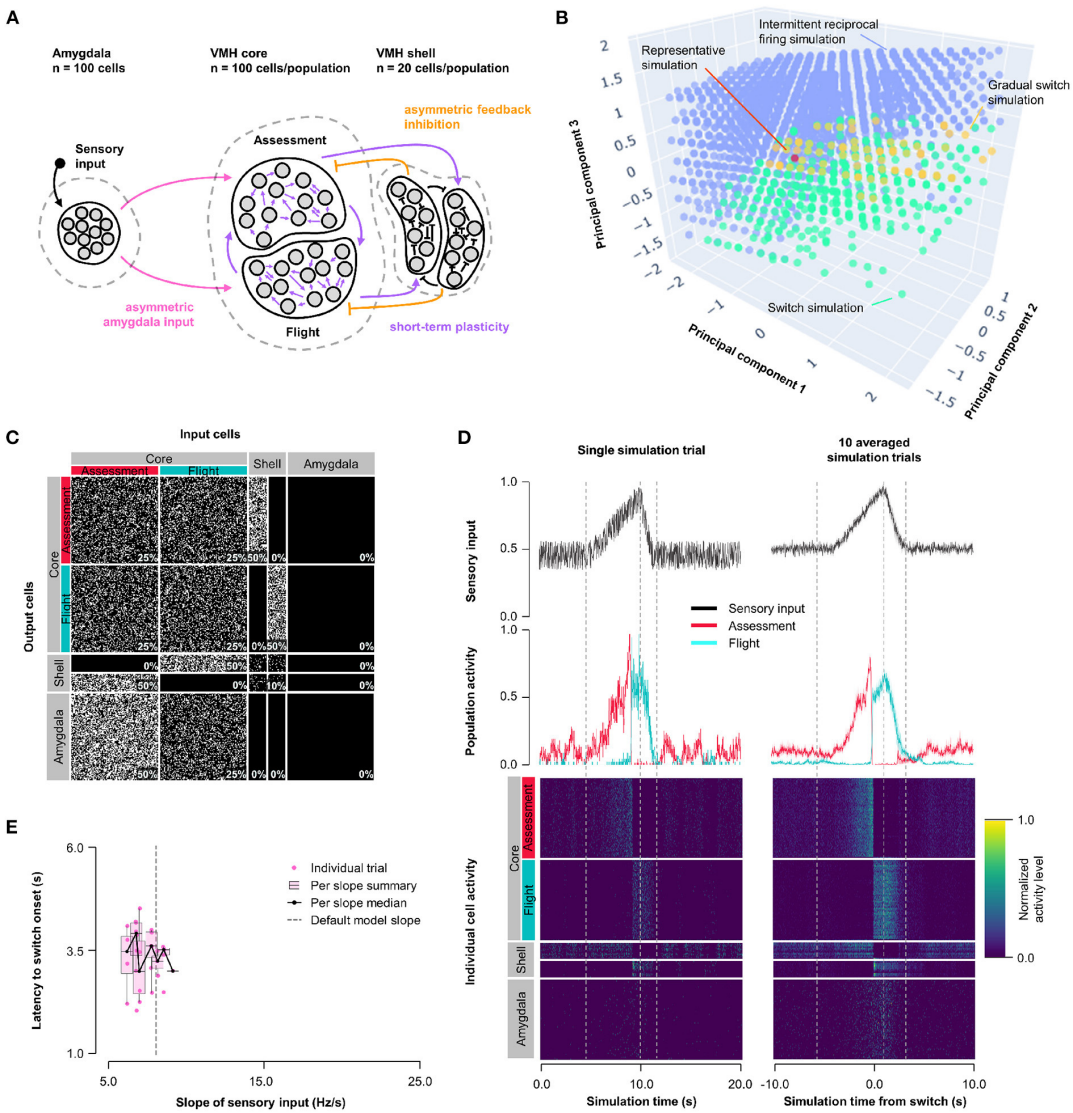
Hybrid model: asymmetric feedback inhibition and short-term plasticity. **(A)** Circuit structure in which the ratio (*r*_*input*_) of the probability of Amygdala-Flight/Amygdala-Assessment connectivity (pink), the ratio (*r*_*feedback*_) of the weights of shell-Assessment/shell-Flight connectivity (orange), and depression and facilitation time constants for short-term plasticity introduced at all excitatory core output connections (purple) were systematically varied. **(B)** Principal component analysis of systematic model exploration identified parameters that showed consistent Assessment/Flight switching (turquoise, see Section 2), gradual switching (result observed in Model 1 simulations, yellow), or intermittent reciprocal firing (result observed in Model 2 simulations, blue). Parameter combinations not falling into either category are not shown. A selected representative switch simulation is indicated in orange. **(C)** Network connectivity matrix of the representative simulation showing connectivity probabilities between all neuron populations. **(D)** Network firing activity from the representative simulation for a single-trial simulation run (left) or a 10-trial averaged simulation run (right). Top row shows sensory input, middle row shows firing patterns of Assessment (red) and Flight (blue) populations, and bottom row shows firing activity of all individual cells, separated by population. All trials were centered around the time the switch occurs before averaging. The vertical dashed lines mark the beginning and end of the assessment and flight phases of the sensory input (see Section 2). **(E)** Plot of sensory input slope vs. latency of switching showing a weak inverse relationship between latency and sensory threshold. Only slope values for which a switch was observed are indicated.

To understand whether the intermittent and unstable firing responses of Flight cells seen in the short-term plasticity model could be mitigated while maintaining its improved switching behavior, we combined the features of Model 1 and 2 into a Hybrid Model. Short-term, pre-synaptic, facilitating plasticity from Model 2 was incorporated into all core neuron outputs, including both core-core and core-shell synapses, in a model with varying Amygdala input density and exclusive feedback inhibition from Flight to Assessment neurons as in Model 1 (Hybrid Model, Figure 3A). We performed parameter exploration of synaptic plasticity parameters (0 ≤ τ_*facilitation*_ ≤ 10, 000 *ms*; 0 ≤ τ_*depression*_ ≤ 1, 000 *ms*), feedback inhibition strength (1 ≤ *r*_*feedback*_ ≤ 20), and Amygdala input asymmetry (0 ≤ *r*_*input*_ ≤ 1); for visualization purposes, principal components (derived from PCA, see Supplementary Figure 4 for component weighting) were used. The Hybrid Model showed intermediate characteristics, including a gradual switch (like Model 1) and intermittent burst firing (like Model 2), but was also able to show switching behavior, even at equal input strength (Figure 3B; representative simulation: τ_*facilitation*_ = 3, 158 *ms*, τ_*depression*_ = 527 *ms*, *r*_*feedback*_ = 4.7, *r*_*input*_ = 0.5, Figures 3C,D; Supplementary Figure 3C). However, the model was not robust to changes in sensory input strengths and showed only a weak trend for sensory input thresholding (Figure 3E).

### 3.5. Model 3: Excitatory rebound

**Figure 4:**
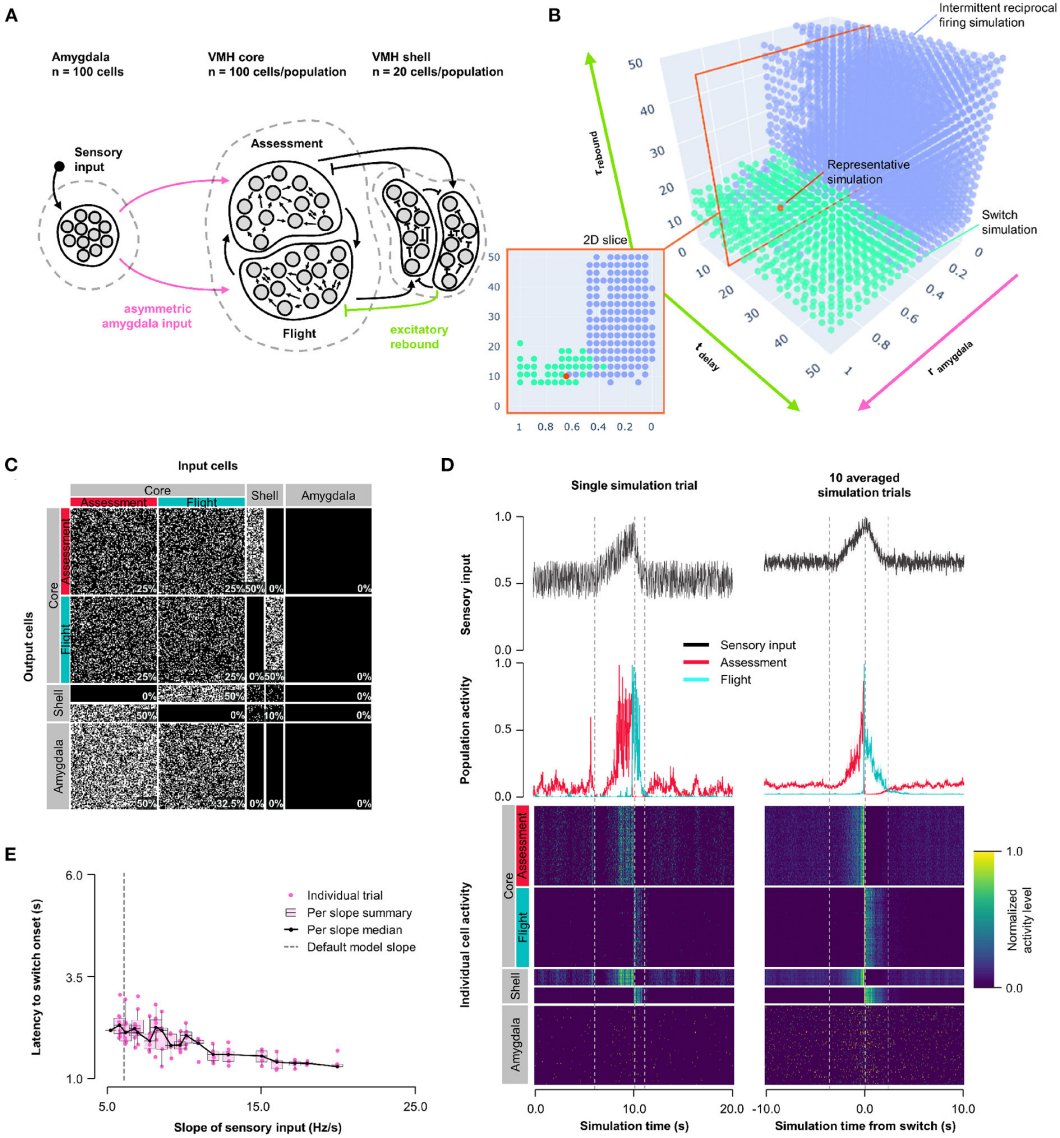
Model 3: Excitatory rebound. **(A)** Circuit structure in which both delay and membrane time constants for excitatory rebound in inhibitory inputs onto Flight neurons (green) as well as the ratio (*r*_*input*_) of the probability of Amygdala-Flight/Amygdala-Assessment connectivity (pink) were systematically varied. **(B)** Systematic model exploration identified parameters that showed consistent Assessment/Flight switching (turquoise, see Section 2) or intermittent reciprocal firing (blue). Parameter combinations not falling into either category are not shown. A selected representative switch simulation is indicated in orange, and the plane in the parameter space containing this simulation (at *t*_*delay*_ = 14 *ms*, also in orange) is shown in the 2D slice in the inset on the bottom left. **(C)** Network connectivity matrix of the representative simulation showing connectivity probabilities between all neuron populations. **(D)** Network firing activity for the representative simulation from a single-trial simulation run (left) or a 10-trial averaged simulation run (right). Top row shows sensory input, middle row shows firing patterns of Assessment (red) and Flight (blue) populations, and bottom row shows firing activity of all individual cells, separated by population. All trials are centered around the time the switch occurred before being averaged. The vertical dashed lines mark the beginning and end of the assessment and flight phases of the sensory input (see Section 2). **(E)** Plot of sensory input slope vs. latency of switching showing an inverse relationship between latency and sensory threshold. Only slope values for which a switch was observed are indicated.

Next, we explored whether other non-linear synaptic mechanisms might facilitate more stable, but nevertheless abrupt, reciprocal activity changes in response to sensory input. VMHvl neurons have been shown to exhibit slow-acting excitatory rebound responses to the optogenetic activation of inhibitory inputs (Wong et al., 2016). Importantly, such rebound responses were restricted to neurons that showed decreased (called Attack- neurons), but not increased (called Attack+ neurons) firing during approach-to-attack behavior (Wong et al., 2016). Although in this study the firing of Attack+/- neurons was not investigated during approach-to-flight behavior, we speculate that these Attack+ and Attack− cells correspond to Assessment+ and Flight+ cells, respectively, because of their similar firing activity responses during social approach (Flight+ cells frequently showed decreased firing during approach; Krzywkowski et al., 2020; Masferrer et al., 2020). Based on these considerations we incorporated slow-acting excitatory rebound responses exclusively at inhibitory inputs onto Flight cells (Model 3, Figure 4A, Supplementary Figure 5A). A systematic exploration of excitatory rebound parameters (0 ≤ *t*_*delay*_ ≤ 50 *ms*; 0 ≤ τ_*rebound*_ ≤ 50 *ms*) and Amygdala input density asymmetry (0 ≤ *r*_*input*_ ≤ 1) identified a relatively narrow range at which Assessment and Flight neurons showed reliable and stable switching behavior (Figure 4B; representative simulation: *t*_*delay*_ = 14 *ms*, τ_*rebound*_ = 10 *ms*, *r*_*input*_ = 0.7; Figures 4C,D, Supplementary Figure 5C). Stable switching was seen regardless of delay time, but only within a narrow range of rebound time constant (τ_*rebound*_∈[7.9, 23.7] *ms*) that matched well to that observed experimentally (Wong et al., 2016). Amygdala input to Flight cells contributed to stabilizing the response dynamics and avoiding intermittent firing, reinforcing the observation that symmetric Amygdala inputs to VMH core neurons is compatible with the emergence of reciprocal firing responses (Supplementary Figure 3D). Again, Model 3 exhibited sensory input thresholding with latency to switch onset varying inversely with sensory input slope (Figure 4E).

### 3.6. Model 4: Non-exclusive feedback inhibition

**Figure 5:**
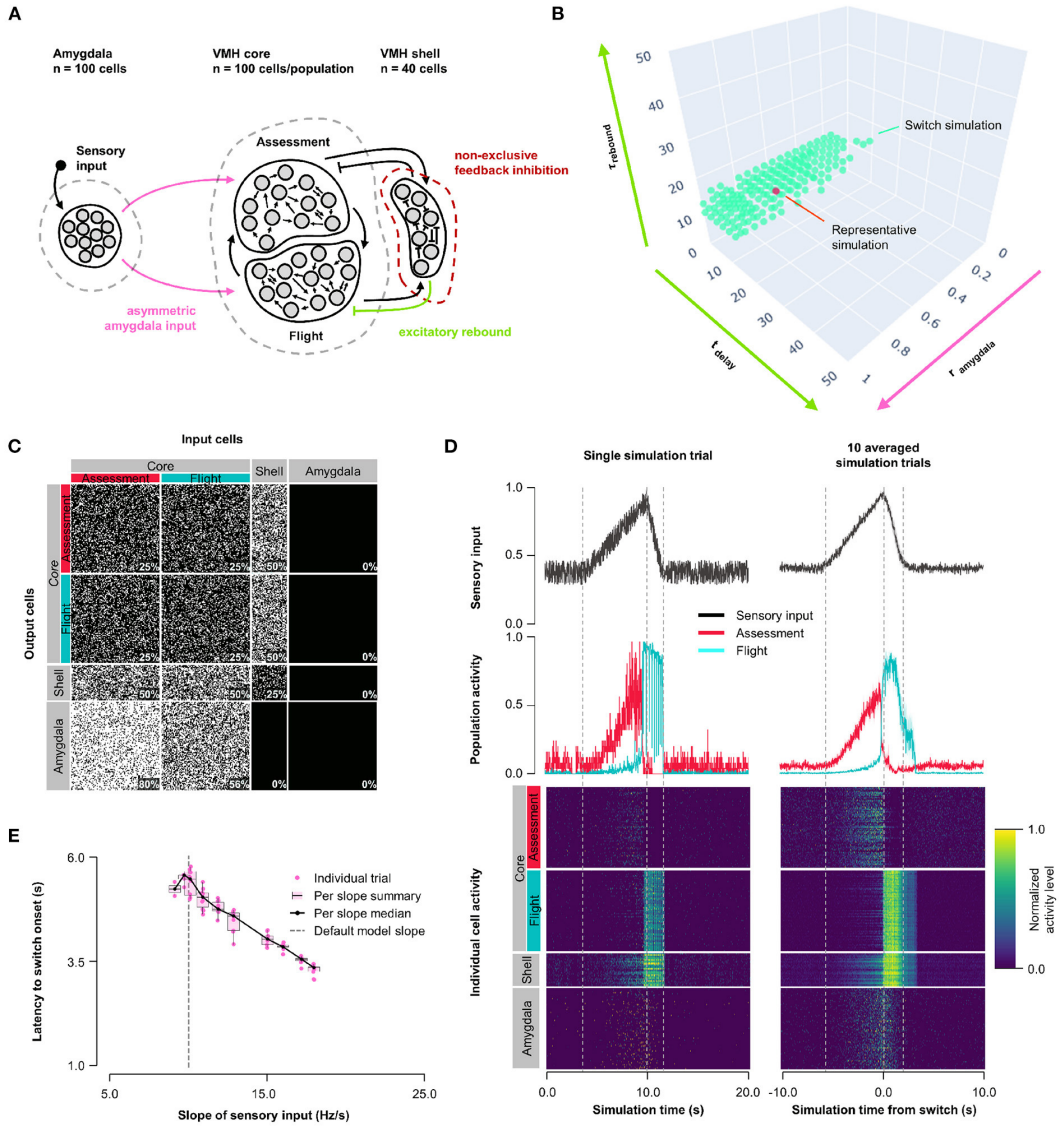
Model 4: Non-exclusive feedback inhibition. **(A)** Circuit structure with shell neurons joined into a single population (dark red) and stochastic core-shell and shell-core connectivity. As in Model 3, the delay time and membrane time constant for excitatory rebound in all inhibitory inputs onto Flight neurons (green) and the ratio (*r*_*input*_) of the probability of Amygdala-Flight/Amygdala-Assessment connectivity (pink) were systematically varied. **(B)** Systematic model exploration identified parameters that satisfy criteria for Assessment/Flight switching (turquoise, see Section 2), where only simulation parameters that satisfy these criteria are shown. A selected representative simulation is indicated (orange). **(C)** Network connectivity matrix of the representative simulation showing connectivity probabilities between all neuron populations. **(D)** Network firing activity for the representative simulation from a single-trial simulation run (left) or a 10-trial averaged simulation run (right). Top row shows sensory input, middle row shows firing patterns of Assessment (red) and Flight (blue) populations, and bottom row shows firing activity of all individual cells, separated by population. All trials are centered around the time the switch occurs before being averaged. The vertical dashed lines mark the beginning and end of the assessment and flight phases of the sensory input (see Section 2). **(E)** Plot of sensory input slope vs. latency of switching showing an inverse relationship between latency and sensory threshold. Only slope values for which a switch was observed are indicated.

Finally, we examined an alternative connectivity for feedback inhibition. In Models 1–3, feedback inhibition was exclusive, with each shell neuron receiving input from either Assessment or Flight neurons and subsequently providing feedback to Flight or Assessment neurons, respectively (Figures 1A–4A). Such exclusive feedback wiring would require high fidelity labeled-line mapping for core-shell-core feedback and could be considered unlikely to occur in nature. In Model 4 we explored an alternative architecture in which core and shell neurons were randomly connected and shell neurons formed a single population (Figure 5A, Supplementary Figure 5B). Systematic exploration of parameter space in this non-exclusive connectivity model using the same conditions as in Model 3 revealed stable Assessment/Flight neuron switching in a limited rebound parameter space (Figure 5B; τ_*rebound*_∈[1.9, 15.9] *ms, t*_*delay*_∈[4.5, 16.5] *ms*; representative simulation: *t*_*delay*_ = 14 *ms*, τ_*rebound*_ = 10 *ms*, *r*_*input*_ = 0.7; Figures 5C,D, Supplementary Figure 5D). Critically, Assessment/Flight neuron switching showed properties similar to those seen *in vivo*, including thresholding and abrupt onset. Moreover, switching persisted even when Amygdala inputs to Assessment and Flight neurons were weighted equally (*r*_*input*_ = 1; Supplementary Figure 3E) and showed sensory input thresholding (Figure 5E). These findings demonstrate that the expression of slow-acting excitatory rebound exclusively in Flight neurons is sufficient for the emergence of distinct Assessment and Flight neurons with thresholded, reciprocal sensory responses in the absence of any differences in their connectivity or intrinsic response properties.

## 4. Discussion

Our computational circuit model of the mouse VMH was constrained by physiological properties derived from *in vitro* slice recordings of VMH neurons (Wong et al., 2016; Yamamoto et al., 2018) and anatomical connectivity derived from *in vivo* tracing studies (Yamamoto et al., 2018; Lo et al., 2019; Yamaguchi et al., 2020). Our aim was to determine whether a relatively simple circuit architecture consisting of excitatory core neurons receiving excitatory sensory input and having excitatory and inhibitory feedback connections (Figures 1A–5A) was sufficient to give rise to neuronal populations exhibiting sensory response patterns similar to the Assessment+ and Flight+ neurons identified by *in vivo* single unit recordings in behaving mice in response to a live predator (Masferrer et al., 2020). We found evidence that such a circuit can support the emergence of distinct neuron populations with reciprocal sensory input responses, and that these populations can exhibit abrupt switching activity that is thresholded to sensory input when excitatory slow-acting rebound properties are applied to feedback inhibition onto a sub-population of core neurons (Model 3–4; Figures 4, 5).

Several conclusions can be drawn from our findings. First, a simple circuit architecture of interconnected excitatory neurons receiving feedback inhibition was sufficient for the emergence of distinct populations of neurons with stable sensory ON and OFF response properties. A previous computational model of VMH circuitry exhibited similar sensory ON/OFF neuron populations (Kennedy et al., 2020) and in the mammalian striatum sparse lateral feedback inhibition has been shown to play a role in stabilizing ON and OFF firing states (Fukai and Tanaka, 1997; Ponzi and Wickens, 2010, 2012, 2013; Moyer et al., 2014) suggesting that feedback inhibition may be a general circuit mechanism to establish stable reciprocal sensory response properties. However, unlike in striatum where the behavioral correlates of sensory ON/OFF neurons are not known, in VMH the switch between Assessment+ and Flight+ neuron firing is tightly associated with an approach-to-avoidance sensory-motor transformation (Masferrer et al., 2020) and thus establishes a testable correlation between behavior and circuit state transitions. Second, our models showed that a simple feedback inhibition architecture can give rise to switching activity that is thresholded to a gradually increasing sensory input. Such thresholding emerged either under specific unbalanced sensory input and feedback inhibition conditions (Model 1, Figure 1) or under balanced connectivity conditions that included unbalanced neuromodulatory rebound excitation (Model 3–4, Figures 4, 5). Thresholded switching offers a mechanism by which accumulating sensory information about threat intensity could trigger the Assessment-to-Flight firing state transition in VMH and potentially engage downstream outputs to initiate escape behavior. It is noteworthy that reciprocally switching Assessment+ and Flight+ neurons are also found in the major upstream and downstream afferents and efferents of VMH, Deng et al. (2016), Evans et al. (2018), Miller et al. (2019), and Masferrer et al. (2020) suggesting that ancient instinctive defense pathways in the brain may have evolved as a hierarchical cascade of circuits that each allow sensory-to-motor transition decisions to emerge along the path from sensory input to locomotor output. A clue to the existence of such distributed sensory-motor decision control arises from the observation that feedback projections from PAG to VMH exert opposing excitatory and inhibitory effects, respectively, on Assessment+ and Flight+ cells in VMH (Masferrer et al., 2020) possibly modulating upstream decision nodes along the sensory-motor pathway. Third, the emergence of Assessment and Flight populations in our models persisted under conditions of equally weighted input and/or feedback connectivity (Model 2–4, Figures 2–5). Such a stochastic wiring of VMH core and shell neurons is consistent with developmental axon pathfinding processes guided by general target attraction and repulsion rules rather than those required for labeled-line connectivity and may provide benefits in the face of evolutionary pressure (Zador, 2019).

As mentioned, a previous computational model of the VMH (Kennedy et al., 2020) was able to show sensory-ON/OFF responses, akin to the Assessment and Flight neurons we observed *in vivo*. However, ON/OFF switching in this model occurred during the decreasing rather than increasing phase of sensory input and thus, while this model demonstrated the capacity for feedback inhibition to drive opposing responses to a circuit input, it did not show sensory input thresholded switching behavior as seen for Assessment and Flight cells *in vivo*. Our findings show that the addition of one or more non-linear circuit transmission features to a simple feedback inhibition model can elicit switching behavior that occurs during the rising phase of sensory input.

Our models make several assumptions that may have biased our results. First, we did not include feedforward inhibition despite anatomical evidence to support its existence in projections from amygdala to VMH (Dong and Swanson, 2004; Padilla et al., 2016; Yamamoto et al., 2018). At present it is not clear what might be the impact of such feedforward inhibition on sensory responding of VMH core neurons, but we can conclude that feedforward inhibition is not necessary for the emergence of distinct Assessment and Flight neurons. Feedforward inhibition by VMH afferents is further complicated by the presence of parallel indirect amygdala input pathways to medial hypothalamus *via* pallidal structures, in particular bed nucleus of stria terminalis (BNST) (Swanson, 2000; Dong and Swanson, 2004, 2006). It has been proposed that such direct and indirect pathways allow for rapidly modulated go-no-go control of behavior as has been described in the basal ganglia (Swanson, 2000). Future models will need to explore the circuit properties afforded by such feedforward control in the medial hypothalamus. In addition, we did not model inhibitory afferents to VMH in our model because of the lack of precise experimental data on the nature of the sensory information encoded by diverse anatomical inputs. Second, we restricted our model to local VMH circuitry and did not attempt to include other nuclei that together form the highly interconnected medial hypothalamic defensive and reproductive systems and their immediate inputs and outputs (Canteras, 2002). For example, it has been shown that VMH projections to anterior hypothalamic nucleus are required for the production of defensive flight, Wang et al. (2015) while the dorsal premammillary nucleus has been shown to support flight from both predators and conspecific threats (Blanchard et al., 2003; Canteras et al., 2008; Motta et al., 2009). Moreover, VMH is known to project to nuclei from which it receives projections (Lo et al., 2019) opening the possibility that multi-synaptic pathways among medial hypothalamic nuclei and their input and output structures could provide important additional circuit mechanisms for supporting the switching of Assessment+ and Flight+ cells in VMH. Finally, we did not attempt to model Assessment- and Flight- cells that are seen during predator exposure *in vivo* (Masferrer et al., 2020) as we consider these to be variants of Flight+ and Assessment+ cells, respectively, that receive varying amounts of sensory input and our major aims was focused on modeling the switch in firing activity shared across VMH cell types.

We interpret the slow-acting excitatory rebound effect observed *in vivo* after sustained inhibition of VMH Attack−, but not Attack+ neurons, Wong et al. (2016) as evidence that Flight+ and Assessment+ neurons may have distinct biophysical properties. In Model 3 we found that conferring excitatory neuromodulatory rebound to inhibitory inputs selectively onto Flight neurons was sufficient for the emergence of distinct Flight and Assessment neuron sensory responses and for their thresholded switching (Figure 4). Because Flight and Assessment neurons were otherwise uniform in this model, our findings demonstrated that slow-acting rebound excitation alone can confer the unique sensory response pattern of Flight neurons. Furthermore, they suggest that the selective expression of an excitatory neuromodulatory receptor on Flight neurons and the release of the cognate neuromodulator from inhibitory shell neurons could be a physiological mechanism sufficient to confer switching on VMH core neurons *in vivo*. VMH expresses several neuropeptides with excitatory metabotropic receptor signaling (Kim et al., 2019; Kennedy et al., 2020); however, to date none of these has been implicated in defensive behavior. Notably, there is precedent in hypothalamic structures for the co-expression of GABA with neuropeptides that are released only under high frequency firing conditions (Hökfelt, 1991; Knobloch et al., 2012). Activation of such dual-neurotransmitter neurons at low frequency would be inhibitory, but at high frequency could be excitatory and provide the excitatory rebound to inhibitory synapses explored in our models.

Several of our findings present hypotheses that can be experimentally tested with existing *in vivo* circuit recording and manipulation tools. Simultaneous *in vivo* single unit electrophysiological recording and optogenetic stimulation (optrodes) could be used to test whether VMH Assessment+ and Flight+ neurons receive balanced amygdala inputs. A similar approach could be used to test whether VMH Assessment+ and Flight+ neurons receive inhibitory inputs from VMH shell with or without excitatory neuromodulatory rebound. Furthermore, our models make specific predictions about the sensory response properties of VMH shell inhibitory neurons that could be tested by cell-type specific recording. In particular, while our initial models (Model 1–3, Figures 1A–4A) predict the presence of Assessment+ and Flight+-like VMH shell neurons, our non-exclusive feedback inhibition model (Model 4, Figure 5A) predicts the existence of VMH shell neurons with a variety of sensory response properties, including hybrid Assessment+ and Flight+ properties (i.e., increasing during both approach and flight). Finally, our exploration of the connectivity properties that most effectively modulate the sensory threshold for Assessment/Flight switching points to a key role for feedback inhibition, rather than excitation in setting the approach-to-flight decision point. Thus, modulatory inputs onto VMH shell such as those that could be achieved by cell-type specific pharmacogenetic inhibition and activation should be highly effective at setting flight threshold and should have a predictable effect on sensitivity to threat. We expect that such experimental testing will in turn uncover new features to be incorporated in future computational models as part of a virtuous computational-experiment cycle.

## Data availability statement

Publicly available datasets were analyzed in this study. This data can be found at: https://gitlab.com/gross-group/vmh-models.

## Author contributions

All simulated models and data analysis were carried out by RR. CG conceived the project and together with HA supervised the project. RR, HA, and CG designed the models and wrote the manuscript. All authors contributed to the article and approved the submitted version.

## Funding

This work was funded by EMBL.

## Conflict of interest

The authors declare that the research was conducted in the absence of any commercial or financial relationships that could be construed as a potential conflict of interest.

## Publisher's note

All claims expressed in this article are solely those of the authors and do not necessarily represent those of their affiliated organizations, or those of the publisher, the editors and the reviewers. Any product that may be evaluated in this article, or claim that may be made by its manufacturer, is not guaranteed or endorsed by the publisher.
