# Supplementary material for: Sensory-thresholded switch of neural firing states in a computational model of the ventromedial hypothalamus
Source: Front Comput Neurosci. 2022 Sep 7;16:964634. doi: 10.3389/fncom.2022.964634 (PMC9491323; doi:10.3389/fncom.2022.964634)
Supplement: Supplementary file 2 [file Data_Sheet_1.pdf]

Supplementary Material

1 SUPPLEMENTARY TABLES AND FIGURES

1.1 Tables

Table S1: All simulation parameters used.

1.2 Figures

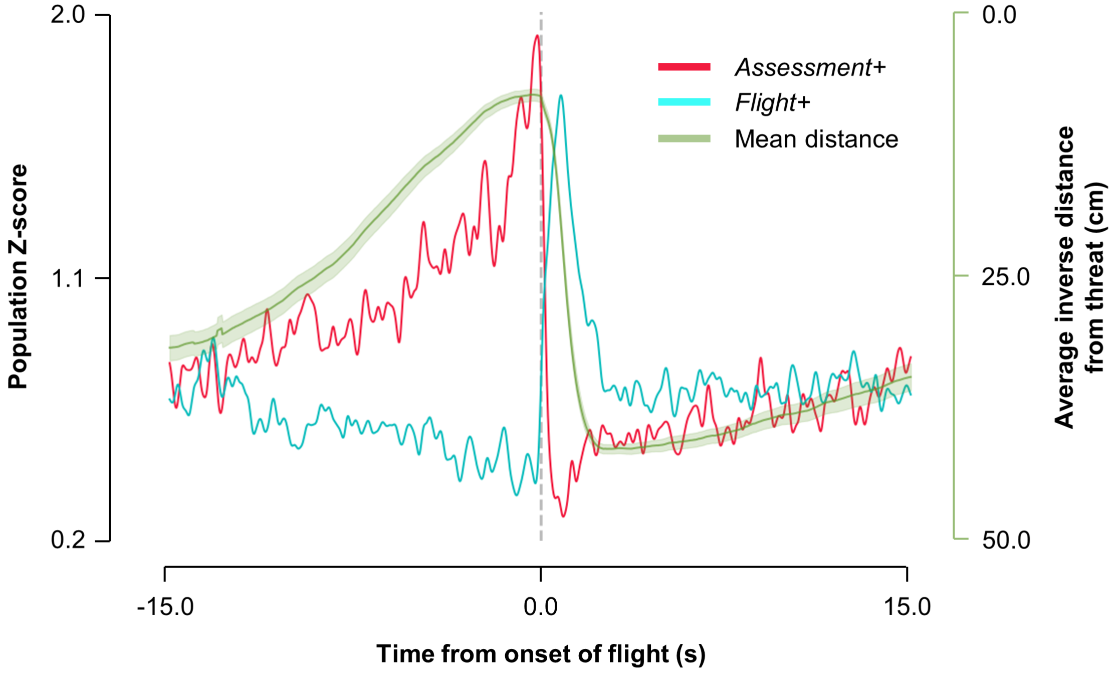

Figure S1. Firing state switch in VMH. In vivo electrophysiology revealed an abrupt and reciprocal switch in firing states in the mouse VMHdm, as seen in the trial-averaged firing of representative Assessment+ (red, average N = 24) and Flight+ (blue, average N = 19) neurons (data reproduced from Masferrer et al. (2020)). An additional trace and its corresponding y-axis (in green) show the mean and standard error of the inverse distance of the mouse to the threat during corresponding approach-avoidance behavior.

REFERENCES

Masferrer, M. E., Silva, B. A., Nomoto, K., Lima, S. Q., and Gross, C. T. (2020). Differential Encoding of Predator Fear in the Ventromedial Hypothalamus and Periaqueductal Grey. *Journal of Neuroscience* 40, 9283–9292. doi:10.1523/JNEUROSCI.0761-18.2020

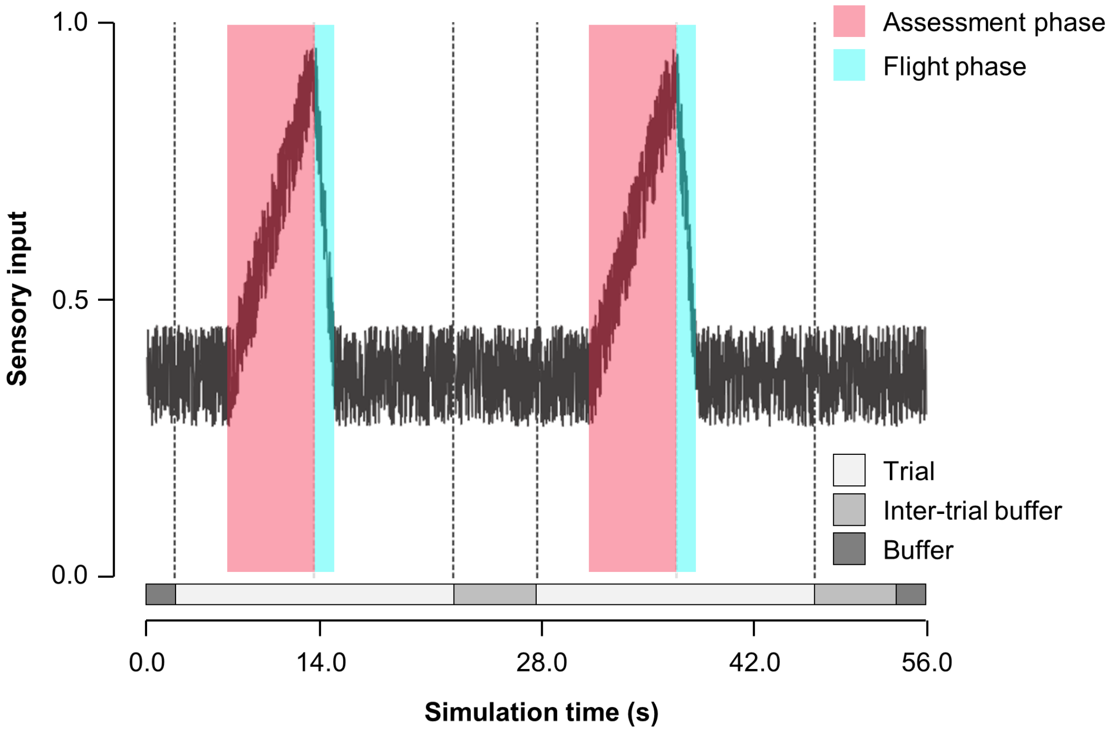

Figure S2. Sensory input function. Noisy sensory input function $\lambda(t)$ used in our models to approximate the fluctuating sensory input mice receive during repeated approach toward and flight away from a stationary threat. Each approach-to-flight trial (two trials are shown here) consists of an approach (red, increasing sensory input) and subsequent flight (blue, decreasing sensory input) phase and is followed by an inter-trial buffer (yellow, stable sensory input) period, with the entire simulation starting and terminating with buffer periods (grey, stable sensory input).

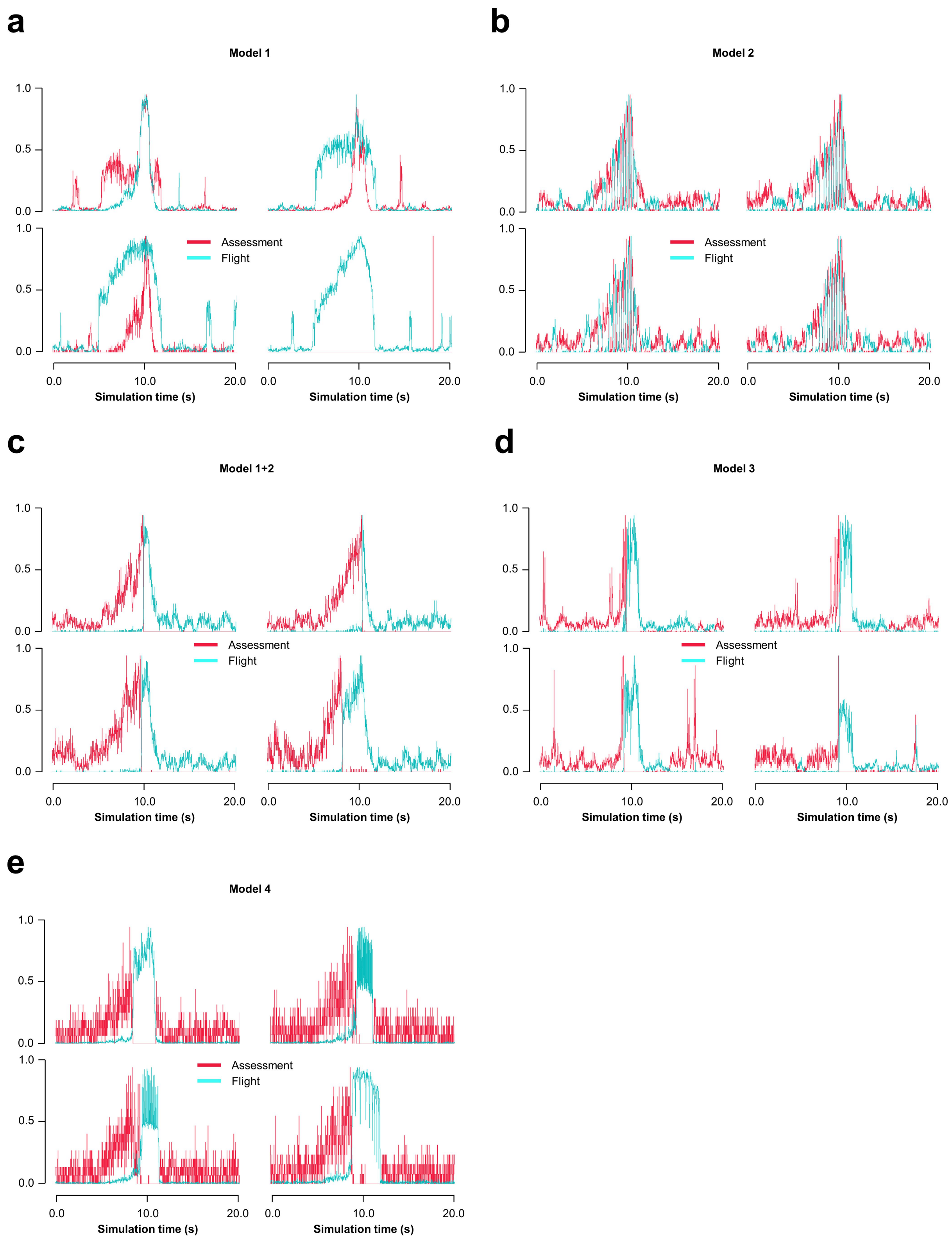

Figure S3. Model results with balanced sensory input. Four example single-trial runs from the parameter exploration of each model with balanced sensory input ($r_{input} = 1$) and varying feedback inhibition, plasticity, and excitatory rebound parameters. Under these conditions Model 1 simulations **(a)** lose their ability to show a switch, while Model 2 simulations **(b)** still show intermittent burst firing and Model 1+2 **(c)**, Model 3 **(d)**, and Model 4 **(e)** simulations still show the switch.

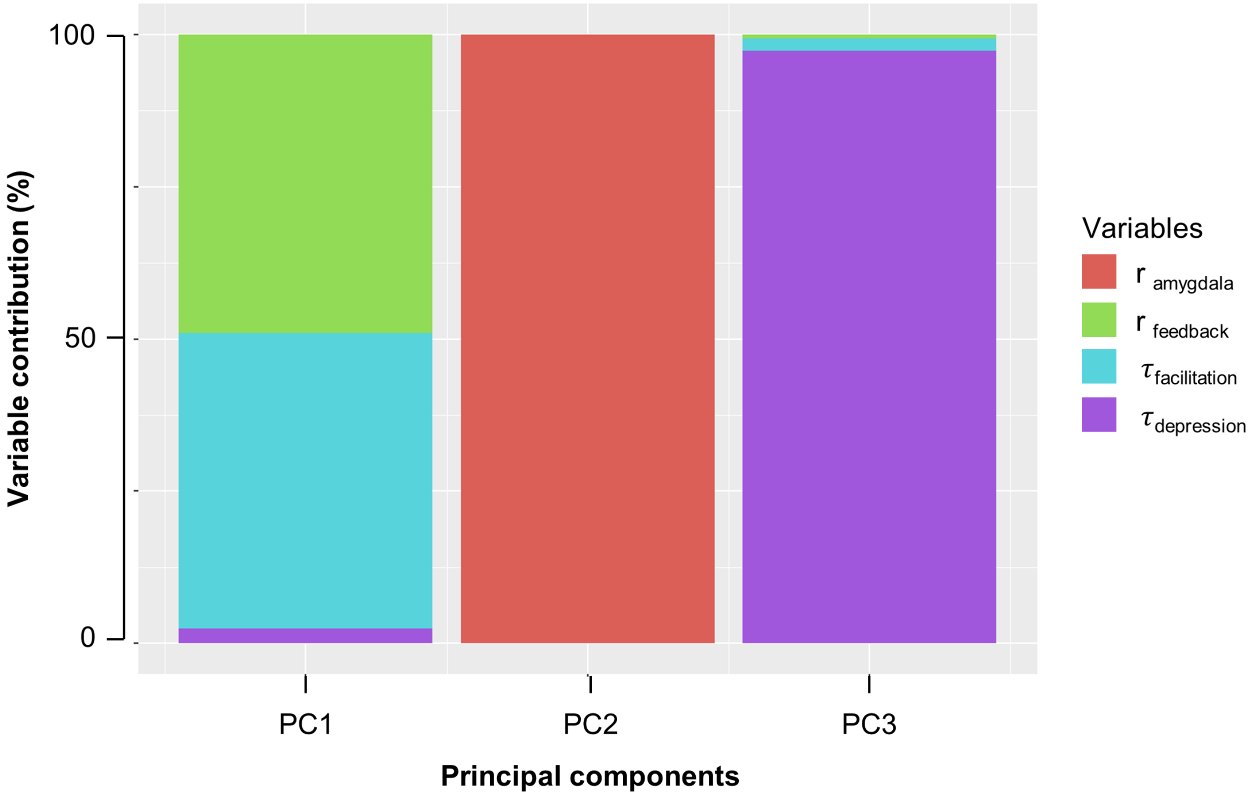

Figure S4. Contribution of each variable to each principal component of the Hybrid Model parameter exploration data, as obtained using principal component analysis.

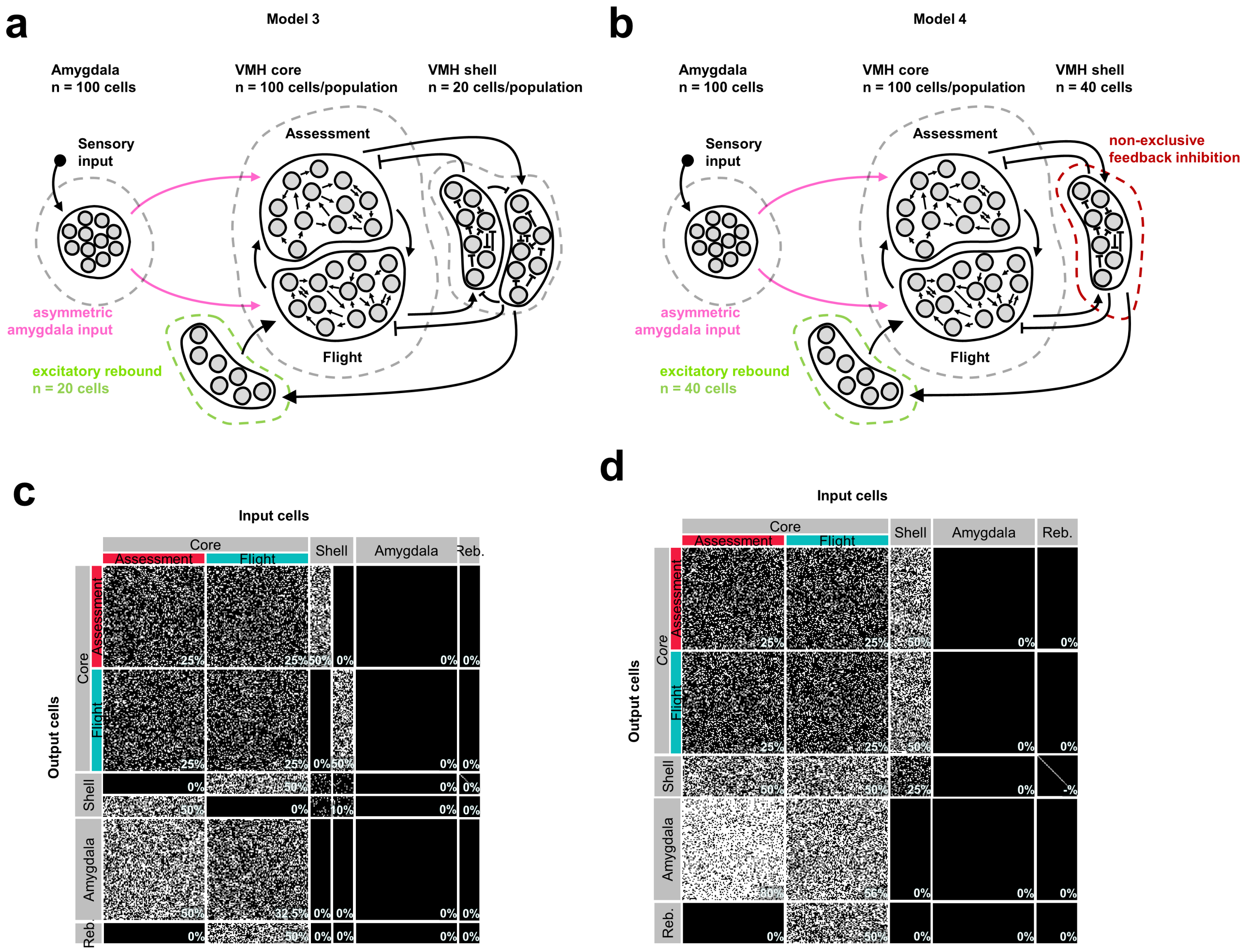

Figure S5. Implementation of excitatory rebound. Circuit structure and connectivity matrix of (**a** and **c**, respectively) Model 3 and (**b** and **d**, respectively) Model 4 showing the added intermediate rebound population required to model inhibitory inputs with slow-acting excitatory rebound.
